# The incidence and prognostic value of silent myocardial scar by late gadolinium enhancement in patients with atrial fibrillation

**DOI:** 10.1186/1532-429X-15-S1-P259

**Published:** 2013-01-30

**Authors:** Tomas G Neilan, Hoshang Farhad, Ravi Shah, Siddique Abbasi, Otavio R Coelho-Filho, John D Groarke, Ciaran J McMullan, Bobby Heydari, Michael L Steigner, Ron Blankstein, Michael Jerosch-Herold, Raymond Y Kwong

**Affiliations:** 1 Cardiology, Brigham and Women's Hospital/Massachusetts General Hospital, Boston, MA, USA; 2 Cardiology, Massachusetts General Hospital, Boston, MA, USA; 3 Radiology, Brigham and Women's Hospital, Boston, MA, USA; 4 Nephrology, Brigham and Women's Hospital, Boston, MA, USA; 5 Internal Medicine, State University of Campinas (UNICAMP), Sao Paulo, Brazil

## Background

Late gadolinium enhancement (LGE) during contrast-enhanced cardiac magnetic resonance (CMR) imaging is a powerful predictor of adverse event. However, there are limited data regarding the incidence and prognostic significance of LGE in patients with atrial fibrillation (AF). Furthermore, while CMR is one of the modalities employed in characterizing pulmonary venous anatomy in patients being considered for radiofrequency ablation therapy, the prognostic significance of an incidental finding of abnormal LGE is unclear. Therefore, our study has 2 aims: 1) to identify the frequency of unanticipated LGE in patients with AF and 2) to determine whether identification of LGE in this population could provide important prognostic information.

## Methods

We performed clinical follow-up on 675 consecutive patients referred for a CMR in preparation for pulmonary vein isolation between September 2005 and March 2011. The clinical end-point of interest was all-cause mortality. Patients with prior myocardial infarction (MI) by clinical history or EKG were excluded. Considering all the significant variables on univariable analysis, we sought the best-overall multivariate models for patient mortality. After excluding patients with MI by clinical history or EKG, all patients were included with all distributions of LGE.

## Results

Fifty five patients (8%) had evidence of a prior MI and were excluded. Of the remaining 620 patients, 73% were male with an average age of 56 years, a BMI of 30±5 kg/m2, a left ventricular (LV) end-diastolic volume of 167±42 mls, an LV mass index of 65±12 gms/m2, and a LV ejection fraction of 56±10%. Of these, 311 patients (50%) had a history of hypertension, 96 (15%) had diabetes, 120 (19%) had sleep apnea, and 156 (25%) had a history of heart failure. LGE was found in 80 patients (13%). There were 61 deaths over a median follow-up period of 42 months. On univariable analysis, age (HR 1.03, CI 1.00-1.06, chi-squared 5.83, P=0.02), diabetes (HR 2.51, CI 1.44-4.36, chi-squared 10.6, P=0.001), heart failure (HR 1.72, CI 1.02-2.90, chi-squared 4.1, P=0.04), the presence of LGE (HR 8.52, CI 4.18-12.2, chi-squared 25.8, P<0.0001), and the extent of LGE (HR 1.15, CI 1.10-1.21, chi-squared 35.6, P<0.0001) provided the strongest association with mortality. In a multivariate model, LGE was the strongest multivariable predictor of mortality. Each adjusted 1% increase in LGE was associated with a 15% increased risk of death.

## Conclusions

In patients with atrial fibrillation and without a prior history of MI, LGE is a frequent finding and is a powerful predictor of mortality. These data support the robust prognostic value of CMR for patients prior to radiofrequency ablation of atrial fibrillation, and support the need for further workup in patients with high-risk findings.

## Funding

This work was supported in part by an American Heart Association Fellow to Faculty Grant (12FTF12060588, TGN), a National Institute of Health T32 Training Grant (T32HL09430101A1, TGN), and project grants (R01HL090634-01A1, MJH; R01HL091157, RYK).

